# Atypical Initial Presentation of Painful Muscle Cramps in a Patient with Amyotrophic Lateral Sclerosis: A Case Report and Brief Review of the Literature

**DOI:** 10.7759/cureus.1837

**Published:** 2017-11-10

**Authors:** Aaron R Kuzel, Muhammad Uzair Lodhi, Intekhab Askari Syed, Mustafa Rahim

**Affiliations:** 1 Department of Emergency Medicine, Lincoln Memorial University-Debusk College of Osteopathic Medicine; 2 Medical Student, Department of Medicine, Raleigh General Hospital, Beckley, Wv; 3 Medical Student, Department of Medicine, Beckley Appalachian Regional Healthcare; 4 Assistant Clinical Professor of Internal Medicine, West Virginia University School of Medicine

**Keywords:** muscle cramps, amyotrophic lateral sclerosis (als), atypical presentation, early diagnosis

## Abstract

Amyotrophic lateral sclerosis (ALS) is a neurodegenerative disorder characterized clinically by progressive muscle weakness that can occur proximally or distally in either the upper or lower extremities. It includes both upper motor neuron signs (spasticity, hyperreflexia, clonus, and Babinski sign) and lower motor neuron signs (atrophy, weakness, and muscle fasciculation). Initial presentation of progressively painful muscle cramps should lead the physician to screen for other signs of amyotrophic lateral sclerosis. We report the case of a 51-year-old male, who presented with dull muscle cramps in the right upper shoulder and arm. After a careful history and physical exam, it was found that patient had both upper and lower motor neuron signs; therefore, a diagnosis of amyotrophic lateral sclerosis was made. Amyotrophic lateral sclerosis should strongly be considered in the differential diagnosis of patients presenting with an atypical initial presentation of progressively painful muscle cramps.

## Introduction

Amyotrophic lateral sclerosis (ALS) is a neurodegenerative disorder that is characterized by degeneration of the anterior horns of the spinal cord, corticospinal tracts, and brain stem. There is a male prevalence of the disease, and it presents as a progressive muscle weakness that can occur proximally or distally in either the upper or lower extremities. The signs seen in a patient include upper and lower motor neuron signs [1]. The upper motor neuron signs usually manifest as spasticity, hyperreflexia, and clonus and can be tested with the Hoffman sign, crossed-adductor sign, and Babinski sign. The lower motor neuron signs usually present with muscle atrophy, weakness, and muscle fasciculations. Rarely, ALS can present as painful muscle cramps. Therefore, practitioners should do a very detailed physical exam in a patient presenting with this atypical presentation. We describe a patient with significant past medical history of bipolar disorder and hyperlipidemia, who developed progressively painful muscle cramps in right upper extremity.

## Case presentation

A 51-year-old male presented to the office with right arm and shoulder pain since November 2016. The patient described the pain as dull, which radiated from the right shoulder to the right arm. He stated that the pain was consistent throughout the day, and he had noticed some weakness in both his right and left arms. The pain started in his right arm and ascended to involve his right shoulder over the year. He reported that nothing exacerbated the pain but did mention that a recent massage relieved most of his pain for a couple of days. The patient denied any injury to the hand or shoulder, weakness in lower extremities, animal exposure, recent travel, or exposure to any rusty penetrating metal. The patient was not in acute distress. His vitals were as follows: blood pressure of 126/86 mm Hg, heart rate of 71 beats per minute, respiratory rate of 16 breaths per minute, and he had no fever. Upon neurological and musculoskeletal examination, the patient had obvious muscle fasciculations noted in bilateral biceps, triceps, and brachioradialis muscles. Bilateral biceps, triceps, and shoulders were tender upon palpation. He had decreased range of motion in the right and left upper extremities of grade 3/5, especially in flexion, extension, and abduction. Decreased grip strength of the right arm in comparison to the left was noted together with right shoulder droop and atrophy, but cranial nerve (CN) XI was intact bilaterally. Reflexes were 4+ with bilateral clonus for the biceps, triceps, and brachioradialis. Two plus patellar and Achilles reflexes were elicited bilaterally. He had a positive Hoffman sign. Finger-to-nose and heel-to-shin tests were intact. He had a non-tender range of motion appreciated in the lower extremities (of grade 5/5). Sensation along the C5-T1 dermatomes was intact to dull and sharp pain recognition. The patient had no ptosis, extraocular muscles were intact, and pupils were equally round and reactive to light. The patient had no noticeable dysdiadochokinesia, dysmetria, or gait abnormalities. We ordered a complete blood count, comprehensive metabolic panel, lipid panel, thyroid studies, vitamin B12, folate, antinuclear antibody (ANA), and creatinine kinase levels. The labs were all within reference ranges. His chest X-ray showed a heart size within normal limits and clear lungs. Magnetic resonance imaging (MRI) lacked any abnormalities with no enhancing periventricular plaques. An electromyography (EMG) study performed indicated decreased muscle strength in both upper extremities from the hand to the shoulder.

## Discussion

Our patient presented with an unusual initial presentation of painful muscle cramps in the right upper extremity; on physical examination, the patient had both upper (hyperreflexia and clonus) and lower (weakness and fasciculations) motor neuron signs, which led to us to consider ALS as a differential diagnosis. To briefly work through the differentials, we ruled out multiple causes of upper motor neuron signs, lower motor neuron signs, and muscular weakness, such as myasthenia gravis, vitamin B12 deficiency, multifocal motor atrophy, polymyalgia rheumatica, and multiple sclerosis. Myasthenia gravis also presents with weakness. However, if the patient had myasthenia gravis, these symptoms would worsen as the day progressed, whereas, in our patient's case, the weakness was consistent throughout the day [2]. In addition, myasthenia gravis would have presented with ptosis, blurry vision, and diplopia. Normal lab values helped us to rule out B12 deficiency as his labs were within limits, and in addition, he didn’t have a loss of temperature sensation or proprioception. Spinal muscle atrophy, which presents with lower motor neuron signs, was also ruled out after a detailed physical exam because the patient, in this case, presented with signs of both upper and lower motor neuron signs. Even though the patient presented with muscle pain and had tenderness on physical examination, normal creatinine kinase, which is specific for inflammatory myositis, helped us to rule out polymyalgia rheumatica. In addition, polymyalgia rheumatica does not present with upper and lower motor neuron signs [3]. Multifocal motor atrophy was not the diagnosis either because even though our patient had upper and lower motor neuron signs, he did not exhibit the parkinsonian, cerebellar, or bulbar affects normally seen in multifocal motor atrophy. In addition to this, patients with multifocal motor atrophy also exhibit autonomic dysfunction in the form of orthostatic hypotension, which our patient did not have [4-5]. Finally, it was difficult to separate multiple sclerosis from amyotrophic lateral sclerosis based on clinical observation alone because multiple sclerosis can have a multitude of inconsistent symptoms, some mirroring those seen in amyotrophic lateral sclerosis. However, given the MRI results were negative for enhancing hyperintense periventricular plaques, we were able to rule out multiple sclerosis [6]. The pathophysiology of muscle cramps in ALS is still not clear; however, a possible explanation could be glutamate excitation, leading to the generation of free radicals and causing spontaneous discharges of motor nerves. Eventually, the spontaneous discharges travel along the nerve trunks to muscle fibers [7]. Most cases of ALS are sporadic, but there has been some testing that has determined mutations in superoxide dismutase 1 (SOD1) and TAR DNA-binding protein (TARDBP) genes [6]. Unlike the patient in this case, ALS patients usually present with a bulbar affect, such as atrophy and weakness of the tongue, leading to dysarthria. The patient with bulbar affect will also have odynophagia and dysphagia [8]. Unfortunately, management of the patient is supportive and palliative care. There is no cure for ALS, and the prognosis for our patient is very poor. If the patient presents with bulbar symptoms, the patient prognosis is two to three years, and if it is limb onset, then the prognosis is three to five years. The only drug that has been shown to extend survival for patients since 1985 is riluzole, which is a glutamate inhibitor [6]. However, a new clinical trial in 2017 on a medication called edaravone (Radicava) had a 33% increase in life expectancy in comparison to the standard of care (Riluzole) [9].

## Conclusions

In our case report, we tried to highlight the variability in the initial presentation of ALS and the importance of a very careful physical exam. Initially, ALS can present as muscle cramps with or without any obvious upper and lower motor neuron signs. However, a detailed physical exam must be performed to catch ALS in early stages so the progression could possibly be slowed down.
